# Small-molecule α-lipoic acid targets ELK1 to balance human neutrophil and erythrocyte differentiation

**DOI:** 10.1186/s13287-024-03711-6

**Published:** 2024-04-08

**Authors:** Yimeng Zhang, Ya Zhou, Xiaohong Li, Xu Pan, Ju Bai, Yijin Chen, Zhenyang Lai, Qiang Chen, Feng Ma, Yong Dong

**Affiliations:** 1 Department of Immunology, School of Basic Medical Sciences, Chengdu Medical College, Xindu Road 783, Chengdu, 610500 China; 2 Center for Stem Cell Research and Application, Institute of Blood Transfusion, Chinese Academy of Medical Sciences & Peking Union Medical College (CAMS & PUMC), Chengdu, China; 3 Sichuan Cord Blood Bank, Chengdu, China

**Keywords:** ALA, ELK1, Neutrophils, Erythrocytes

## Abstract

**Background:**

Erythroid and myeloid differentiation disorders are commonly occurred in leukemia. Given that the relationship between erythroid and myeloid lineages is still unclear. To find the co-regulators in erythroid and myeloid differentiation might help to find new target for therapy of myeloid leukemia. In hematopoiesis, ALA (alpha lipoic acid) is reported to inhibit neutrophil lineage determination by targeting transcription factor ELK1 in granulocyte-monocyte progenitors via splicing factor SF3B1. However, further exploration is needed to determine whether ELK1 is a common regulatory factor for erythroid and myeloid differentiation.

**Methods:**

In vitro culture of isolated CD34^+^, CMPs (common myeloid progenitors) and CD34^+^ CD371^–^ HSPCs (hematopoietic stem progenitor cells) were performed to assay the differentiation potential of monocytes, neutrophils, and erythrocytes. Overexpression lentivirus of long isoform (L-ELK1) or the short isoform (S-ELK1) of ELK1 transduced CD34^+^ HSPCs were transplanted into NSG mice to assay the human lymphocyte and myeloid differentiation differences 3 months after transplantation. Knocking down of SRSF11, which was high expressed in CD371^+^GMPs (granulocyte-monocyte progenitors), upregulated by ALA and binding to ELK1-RNA splicing site, was performed to analyze the function in erythroid differentiation derived from CD34^+^ CD123^mid^ CD38^+^ CD371^–^ HPCs (hematopoietic progenitor cells). RNA sequencing of L-ELK1 and S-ELK1 overexpressed CD34^+^ CD123^mid^ CD38^+^ CD371^–^ HPCs were performed to assay the signals changed by ELK1.

**Results:**

Here, we presented new evidence that ALA promoted erythroid differentiation by targeting the transcription factor ELK1 in CD34^+^ CD371^–^ hematopoietic stem progenitor cells (HSPCs). Overexpression of either the long isoform (L-ELK1) or the short isoform (S-ELK1) of ELK1 inhibited erythroid-cell differentiation, but knockdown of ELK1 did not affect erythroid-cell differentiation. RNAseq analysis of CD34^+^ CD123^mid^ CD38^+^ CD371^–^ HPCs showed that L-ELK1 upregulated the expression of genes related to neutrophil activity, phosphorylation, and hypoxia signals, while S-ELK1 mainly regulated hypoxia-related signals. However, most of the genes that were upregulated by L-ELK1 were only moderately upregulated by S-ELK1, which might be due to a lack of serum response factor interaction and regulation domains in S-ELK1 compared to L-ELK1. In summary, the differentiation of neutrophils and erythrocytes might need to rely on the dose of L-ELK1 and S-ELK1 to achieve precise regulation via RNA splicing signals at early lineage commitment.

**Conclusions:**

ALA and ELK1 are found to regulate both human granulopoiesis and erythropoiesis via RNA spliceosome, and ALA-ELK1 signal might be the target of human leukemia therapy.

**Supplementary Information:**

The online version contains supplementary material available at 10.1186/s13287-024-03711-6.

## Background

Anemia is relatively common in patients with chronic myeloid leukemia (CML) [1], acute myeloid leukemia (AML), and myelodysplastic syndromes (MDS) [2]. Aplastic anemia can develop into MDS and AML, and is associated with poor outcomes [3, 4], and can also co-occur in patients with CML [1, 5] and AML. In addition, several case reports show that Fanconi anemia can transform into AML [6–8]. Late anemia [9], late chronic anemia [10], and aplastic anemia [11] can occur in patients with CML treated with imatinib; in such patients erythropoietin treatment may be beneficial [12]. Impaired erythrocyte differentiation is observed in patients with MDS [13] and AML [14]. The AML1–ETO fusion protein associated with M2 AML inhibits normal hematopoiesis and blocks erythrocyte differentiation [15]. Together, this evidence indicates that there exist some factors regulating both erythroid cell and myeloid cell differentiation, and these factors could be targets for myeloid leukemia therapies.

Both erythrocyte and neutrophil differentiation are affected by levels of reactive oxygen species (ROS). In bone marrow, myeloid cell-derived ROS promote emergency granulopoiesis [16]; common myeloid progenitors (CMPs) with higher ROS levels differentiate into myeloid cells and CMPs with lower ROS levels differentiate into erythroid cells [17]. We hypothesize that factors exist downstream of ROS-related signals that help balance the differentiation of erythroid cells and myeloid cells. For example, SF3B1 regulates erythroid-cell proliferation and maturation [18, 19], and balances the differentiation of neutrophils and monocytes [20] through the transcription factor ELK1, which is associated with AML [21] and is regulated by the antioxidant α-lipoic acid (ALA). ELK1 is upregulated in many other cancers, such as cervical cancer [22], pancreatic cancer [23], thyroid cancer [24], colorectal cancer [25], and breast cancer [26]. Suppression of ELK1 inhibits progression of thyroid cancer [24], pancreatic cancer [27], breast cancer [28], and cervical cancer [29] in in vitro and in vivo models. ELK1 has several domains including DNA-binding domains and a serum response factor (SRF) interaction and regulation domain (a phosphorylation domain), and the various phosphorylation sites of ELK1 play different roles in regulating cell proliferation and cell differentiation [30]. ELK1 gene produces a small protein containing 95 amino acids (S-ELK1) (Accession: NM_001257168.1), which only contains the DNA binding domain and part of phosphorylation sites compared to the full-length protein (L-ELK1). Furthermore, the different isoforms of ELK1 could be regulated by RNA splicing, for example ALA down-regulates ELK1 to block neutrophil lineage differentiation via SF3B1 [20].

To further understand the links between the differentiation of cells of erythroid lineage and neutrophil lineage and identify new targets for myeloid leukemia therapies that do not produce anemia, our work focuses on the function of ALA in regulating lineage-specific differentiation. Our previous studies show that ALA promotes the expansion of hematopoietic stem cells [31] and blocks neutrophil differentiation by targeting ELK1 through regulating RNA splicing [20]. Here, we discovered a new function of ALA in promoting erythroid-cell differentiation by targeting ELK1. We found that overexpression of ELK1 inhibited erythrocyte differentiation, while ELK1 knockdown did not affect erythrocyte differentiation, indicating that ELK1 negatively regulates erythrocyte differentiation. Overall, our results suggest that ELK1 is a potential target for treatments of myeloid leukemia.

## Material and methods

### Cell culture

All hematopoietic stem progenitor cells (HSPCs) are isolated from cord blood prepared by the Sichuan Cord Blood Bank.

To reveal the function of ALA in the differentiation of human hematopoietic lineage cells, CD34^+^ hematopoietic stem progenitor cells (HSPCs) were sorted and cultured in Iscove modified Dulbecco media (IMDM) supplied with eight cytokines (10 ng/mL SCF, 10 ng/mL FLT3L, 10 ng/mL TPO, 10 ng/mL IL-3, 10 ng/mL IL-6, 10 ng/ mL GM-CSF, 20 ng/mL G-CSF, and 2 U/mL EPO; all cytokines were purchased from PeproTech). Monocytes, neutrophils, and erythrocytes were detected after 14 days of in vitro differentiation. Treatment of cells with 50 μg/mL ALA was performed as previously described [20].

To assay the potential of different progenitors to differentiate into erythrocytes, progenitor cells were sorted and cultured in IMDM supplemented with eight cytokines (10 ng/mL SCF, 10 ng/mL Flt3l, 10 ng/mL IL-3, 10 ng/mL IL-6, 10 ng/mL GM-CSF, 20 ng/mL G-CSF, 5 ng/mL TPO, and 2 U/mL EPO) for 8 days, and then 200 μg/mL transferrin was added to the medium to facilitate further differentiation. CD14^+^ monocytes, CD14^–^ CD66b^+^ neutrophils, and CD11b^–^ CD71^+^ GPA^+^ erythrocytes were detected on day 14 of in vitro culture.

Hematopoietic progenitor cells at different stages of erythrocyte differentiation derived from CD34^+^CD45RA^–^ HSPCs were sorted on day 4 and cultured with StemSpan SFEM II media (Stem Cell Technologies, cat #09655) supplemented with 10 ng/mL SCF, 10 ng/mL FLT3L, 2 U/mL EPO, and 10 ng/mL IL-3.

### Construction of gene-expressing plasmids and shRNA-expressing plasmids

The cDNA of L-ELK1 and S-ELK1 are cloned into PCCL-MND-PGK-EGFP vector to generate the PCCL-ELK1-GFP, PCCL-S-ELK1-GFP overexpression vectors and shRNA expression vectors are cloned using Plko.1-EGFP-Puro (#FH1717, FENGHUISHENGWU Co., Ltd, China). The target sequence of ELK1-shRNA-A (L-ELK1 and S-ELK1) is GCCTTGCGGTACTACTATGACA and target sequence of ELK1-shRNA-L is CCAAACCTGAAATCGGAAGAGC. To knocking down of SRSF1, SRSF3, SRSF11, the target sequences of SRSF3 and SRSF11 are: TGGAACTGTCGAATGGTGAAA (SRSF3), and GAGCTTTGATAGTCGTACCAT (SRSF11). All these expression vectors were constructed as previously described [20].

### Lentiviral transduction and assay of erythrocyte, neutrophil or monocyte differentiation potential

Lentivirus preparation of L-ELK1, S-ELK, ELK1-shRNA-A, ELK1-shRNA-L, SRSF3-shRNA and SRSF11-shRNA lentiviral transduction of CD34^+^ HSPCs, and the assay to determine the erythrocyte differentiation potential of the sorted cells (CD123^+^CD71^–^ progenitors, CMPs, and lentiviral-transduced GFP^+^ CD371^–^ CD34^+^ HSPCs), were performed as previously described [20].

### Mice and transplantation

Mx1-Cre (C57BL/6, CD45.2+) were purchased from Cyagen Biosciences Inc. Elk1^LSL/+^ (Tdtomato expression sequence was linked to Elk1 (long isoform transcripts) expression sequence before the stop codon) mice were generated by direct targeting the zygotes of C57BL/6 mice by Crisp-Cas9 at the ROSA26 locus, and subsequently bred with Mx1-Cre to generate Elk1^LSL/+^Mx1-Cre (Mx1-Elk1). To induce the overexpression of Elk1, 200 μg Polyinosinic–polycytidylic acid sodium salt (PolyIC) (Sigma, P1530-100MG) was injected into seven Mx1-Elk1 and seven littermate Ctrl mice through intraperitoneal injection every other day for three times. Seven days later, the PolyIC treated mice are anesthetized by the mixture of oxygen and isoflurane using the Matrx VMR Small Animal Anesthesia Machine, and then peripheral blood was obtained from the retro-orbital venous sinus. Subsequently, all these mice are euthanized by taking carbon dioxide (CO_2_) in a closed environment, and the bone marrow cells are isolated from these mice. The bone marrow single nucleus cells are stained with the mixture of antibodies to analyze different types of hematopoietic cells. Cytometry assay was performed to analyze erythrocyte, neutrophil, monocyte, T cell, B cell and myeloid progenitors 7 days post the last treatment of PolyIC.

All the recipient mice are anesthetized by the mixture of oxygen and isoflurane using the Matrx VMR Small Animal Anesthesia Machine. Subsequently, twenty thousand GFP^+^ CD34^+^ HSPCs transduced with L-ELK1-OE, S-ELK1-OE, were transplanted into 1.0 Gy-irradiated female M-NSG mice (NOD-Prkdcscid Il2rgem1Smoc) (provided by Shanghai Model Organisms Center, Inc.) via retro-orbital vein injection. CD34^+^HSPCs derived CD19^+^CD11b^–^ B lymphocytes, CD19^–^ CD11b^+^ myeloid cells, CD15^–^ CD14^+^ monocytes and CD15^+^CD14^–^ neutrophils, CD123^high^ dendritic cells and CD123^mid^ CD34^+/low^ CD15^+^ CD64^+^ neutrophil progenitors were detected 3 months after transplantation. All the recipient mice are euthanized by taking carbon dioxide (CO_2_) in a closed environment, and the bone marrow cells are isolated from these mice. The bone marrow single nucleus cells are stained with the mixture of antibodies to analyze different types of hematopoietic cells described above. All the mice were housed in EVC Barrier Ventilation System. All animal experiments were approved by the Institutional Ethics Review Committee of the Chengdu Medical College and Institutional Ethics Review Committee of Institute of Blood Transfusion (IERC-IBT).

### Bulk cell RNAseq

Two thousand lentiviral-transduced GFP^+^ GFP^+^ CD123^+^ CD38^+^ CD34^+^ CD371^–^ HPCs expressing GFP-Ctrl, L-ELK1-OE, or S-ELK1-OE were sorted into a 1.5 mL tube containing 200 μL 0.5% BSA-DPBS solution. The dsDNA and cDNA libraries were generated as previously described [32] and sequenced using an Illumina NovaSeq6000 system (Novogene Co., Ltd.). The cleaned fastq data were aligned to the HG38 human reference genome using HISAT2. Raw counts of all RNA-seq samples are calculated by featureCount from the sam files generated by HISAT2. Normalized counts are generated by R packages of DESeq2 from raw counts file, and gplots and clusterProfiler, were used to analyze differentially expressed genes, and gene ontology (GO) enrichment as described previously [20].

### ELK1-RNA binding splicing factors analysis

To analyze the ELK1-RNA binding splicing factors, according to the sequence of S-ELK1 and L-ELK1, we synthesized two 5′-biotin-modified RNA around the splicing site: AGUUUGUGUCCUACCCUGAG, CACUUCUGGAGCACCCUGAG (5′–3′). RNA pulldown and mass spectrometry assay were performed to identify the ELK-RNA binding splicing factors as we previously reported [20].

### RT-PCR and immunofluorescence assay of L-ELK1 and S-ELK1

To detect the expression level of L-ELK1 and S-ELK1 in CD34^+^ hematopoietic stem progenitor cells with SRSF11 knocking down, CD34^+^GFP^+^ cells were sorted, and dsDNA was generated as same as the method for RNAseq described above. The primer pair1 was used to detect both L-ELK1 and S-ELK1: GGGGCTACGCAAGAACAAGA (Forward primer), GCTCACCTTGCGGATGATGT (Reverse primer), primer pair2 was used to detect only L-ELK1: ACATCATCCGCAAGGTGAGC (Forward primer), GGAGGTAACAGACACCTCTG (Reverse primer). The primer pair for B2M is GAGGCTATCCAGCGTACTCCA (Forward primer), CGGCAGGCATACTCATCT TTT (Reverse primer). The RT-PCR reactions were performed in a Thermal cycler (Bio-Rad). The expression value of ELK1 or L-ELK1was calculated using minimal cycle threshold (Ct) values normalized to expression of the housekeeping gene B2M Relative expression value of (L + S)ELK1 or L-ELK1 was calculated as 2^−(Ct value of B2M minus Ct value of L−ELK1 or (L+S)ELK1)^. Immunofluorescence staining assay of the protein expression level of L-ELK1 by using anti-ELK1 C-terminus antibodies (Affinity, AF6212, only indicates the L-ELK1) was performed as previous described [20].

### Data availability and statistical analysis

All data supporting this study are available from the corresponding author upon request. All the RNA-Seq data are deposited in the GEO dataset with the accession number GSE217887 and GSE184864. R packages of DESeq2 and clusterProfiler were used to statistically analyze RNAseq data. Differential expression gene (DEG) of RNAseq data was screened by DESeq2 with the threshold of p.adj < 0.05, and foldchange > 1.5. GO enrichment analysis of indicated DEG list was performed by clusterProfiler. All the flowcytometry data are analyzed by FlowJo X software. All quantitative data are shown as the mean ± standard deviation (SD). Statistical analysis was performed using unpaired Student’s t-tests in GraphPad Prism (GraphPad Software).

### Checklist statement

The work has been reported in line with the Additional file 2: ARRIVE guidelines 2.0.

## Results

### ALA promotes the early differentiation of erythroid progenitors derived from CMPs

**Fig. 1:**
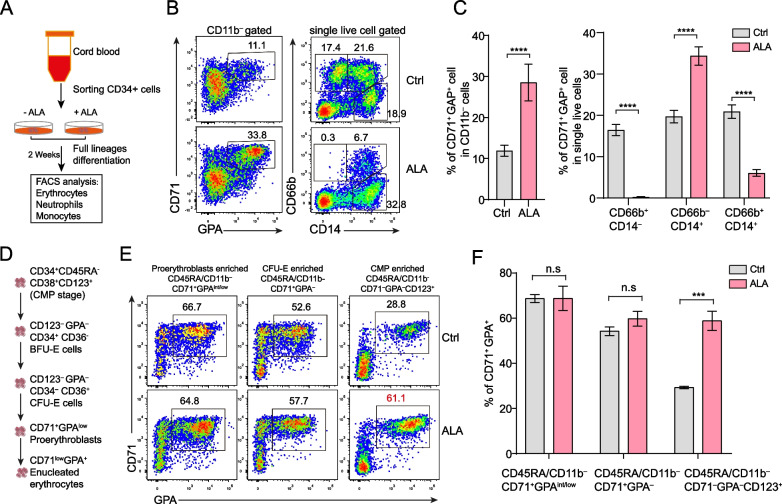
ALA promotes erythroid differentiation of CD123^+^ CD45RA^–^ CD71^–^ progenitors. **A** A schematic of the design of the experiments used to analyze the impact of 50 μg/mL ALA on hematopoietic-cell differentiation. **B** Flow cytometry assays of CD11b^–^ CD71^+^ GPA^+^ erythrocytes, CD66b^+^ CD14^–^ neutrophils, and CD66b^–^ CD14^+^ monocytes were performed on day 14 in the presence or absence of ALA treatment. **C** Statistical analysis of the percentages of  CD71^+^ GPA^+^ erythrocytes in CD11b^–^ cells, CD66b^+^ CD14^–^ neutrophils, CD66b^+^CD14^+^ monocytes and CD66b^–^ CD14^+^ monocytes in single live cells derived from CD34^+^ HSPCs. **D** A schematic plot shows the surface markers changing characteristic of erythroid differentiation from CMP. **E** Cytometry flow assays of CD71^+^ GPA^+^ erythrocytes derived from CD45RA^–^ CD11b^–^ CD71^+^ GPA^–^ CFU-E enriched progenitors, CD45RA^–^ CD11b^–^ CD71^+^ GPA^int/low^ proerythroblasts enriched progenitors, and CD45RA^–^ CD11b^–^ CD123^+^ CD71^–^ GPA^–^ CMP enriched progenitors were performed on day 14 in the presence or absence of ALA treatment. **F** Statistical analysis of the percentage of CD71^+^ GPA^+^ erythrocytes derived from CD45RA^–^ CD11b^–^ CD71^+^GPA^–^ progenitors, CD45RA^–^ CD11b^–^ CD71^+^ GPA^+^ erythrocyte progenitors, and CD45RA^–^ CD11b^–^ CD123^+^ CD71^–^ GPA^–^ CMP-like progenitors were performed on day 14 in the presence or absence of ALA treatment. All the progenitors were sorted from ex vivo cultures of CD34^+^ HSPCs on day 4. The data in the bar graphs in panels (C, F) represent the mean ± SD. An unpaired Student’s t-test (2-tailed) was performed. N = 3–4 replicates; n.s: no significance, *P < 0.05, **P < 0.01, ****P < 0.0001

Our previous reports show that ALA improves the expansion and function of HSPCs [31], and blocks the differentiation of neutrophils derived from HSPCs [20] in ex vivo culture. To further understand the role of ALA in erythropoiesis, we performed in vitro differentiation culture of HSPCs treated with ALA (Fig. 1A). The results showed that ALA significantly increased the production of CD71^+^ GPA^+^ erythrocytes (28.5 ± 2.0% vs. 11.9 ± 0.5%) gated from CD11b^–^ cells. At the same time, ALA increased the production of CD66b^–^ CD14^+^ monocytes, while decreased the production of CD66b^+^CD14^–^ neutrophils [20] and CD66b^+^CD14^+^ proinflammatory monocytes [33] or antigen-presenting cells (APCs)-like tumor associate neutrophil like cells [34] derived from CD34^+^ HSPCs (Fig. 1B, C). We then further investigated the stages at which ALA promotes erythrocyte differentiation. First, we found that ALA promoted the erythrocyte differentiation of CD71^–^ CD105^–^ GPA^–^ cells (Additional file 1: Fig. S1A). We sorted CD71^+^ CD105^+^ CD41^–^ cells and CD71^–^ CD105^–^ CD41^+^ cells for further erythrocyte differentiation (Additional file 1: Fig. S1B). The results showed that ALA promoted the erythrocyte differentiation of CD71^–^CD105^–^CD41^+^ cells but not the differentiation of CD71^+^CD105^+^ cells (Additional file 1: Fig. S1C). Thus, we thought that ALA might promote the commitment of erythrocyte progenitors at earlier stages. Then, we sorted proerythroblasts (CD11b^–^CD45RA^–^CD71^+^GPA^int/low^), CFU-E enriched cells (CD11b^–^CD45RA^–^CD71^+^GAP^–^) [35] and CD11b^–^CD45RA^–^CD71^–^ GPA^–^CD123^mid^ CMP enriched cells (Fig. 1D) derived from HSPCs at day 5 (Additional file 1: Fig. S1D) for further culture. A significantly higher number of erythrocytes were detected from CD45RA^–^CD11b^–^CD71^–^ GPA^–^CD123^+^ CMP enriched cells treated with ALA, but not from CD45RA^–^ CD11b^–^CD71^+^ GPA^int/low^ and CD45RA^–^CD11b^–^CD71^+^GPA^–^ cells treated with ALA (Fig. 1E, F).

**Fig. 2:**
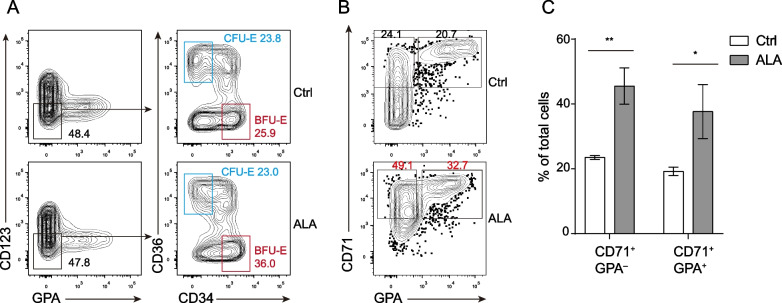
ALA promotes early erythroid differentiation of CMPs. **A** Cytometry flow assays of CD123^–^ GPA^–^ CD34^+^ CD36^–^ BFU cells and CD123^–^ GPA^–^ CD34^–^ CD36^+^ CFU cells derived from CMPs were performed on day 4 in the presence or absence of ALA. **B** Cytometry flow assay of CD71^+^ GPA^–^ progenitors and CD71^+^GPA^+^ erythrocytes derived from CMPs were performed on day 12 in the presence or absence of ALA. **C** Statistical analysis of the percentages of CD71^+^ GPA^–^ progenitors and CD71^+^ GPA^+^ erythrocytes in the presence and absence of ALA were performed with data collected on day 12. The data in the bar graphs in panel (C) are presented as the mean ± SD. An unpaired Student’s t-test (2-tailed) was performed. N = 3–5 replicates; *P < 0.05, **P < 0.01

Given that ALA greatly improved the production of erythrocytes derived from the CD11b^–^ CD45RA^–^CD71^–^ GPA^–^CD123^+^ CMP enriched progenitors, we hypothesized that ALA promoted the commitment of CMPs to erythroid progenitors. Thus, we analyzed erythroid progenitors (CFU-E and BFU-E; burst-forming units) derived from CMPs on day 4 of ALA treatment. The results indicated that ALA significantly improved the production of CD123^–^ GPA^–^ CD36^–^ CD34^+^ BFU-E cells but not the production of CD123^–^ GPA^–^ CD36^+^ CD34^–^ CFU cells at day 4 (Fig. 2A) and improved the production of CD71^+^ GPA^–^ CFU-E enriched progenitors and CD71^+^ GPA^+^ erythrocytes (Fig. 2B, C) on day 12. Additionally, the expression level of GPA was significantly lower under the treatment of ALA than Ctrl (Additional file 1: Fig. S2A). This result pointed out that ALA might also inhibit the immune function of erythrocytes via GPA receptor, just like ALA down regulated the infection related surface markers CD11b, CD54, CD812 (Additional file 1: Fig. S2B) and IL-8 in neutrophils and monocytes (Additional file 1: Fig. S2C, D). Together, the above data indicate that ALA promoted the differentiation of CMPs toward an erythroid lineage at early stage.

### ELK1 inhibits erythroid lineage differentiation

**Fig. 3:**
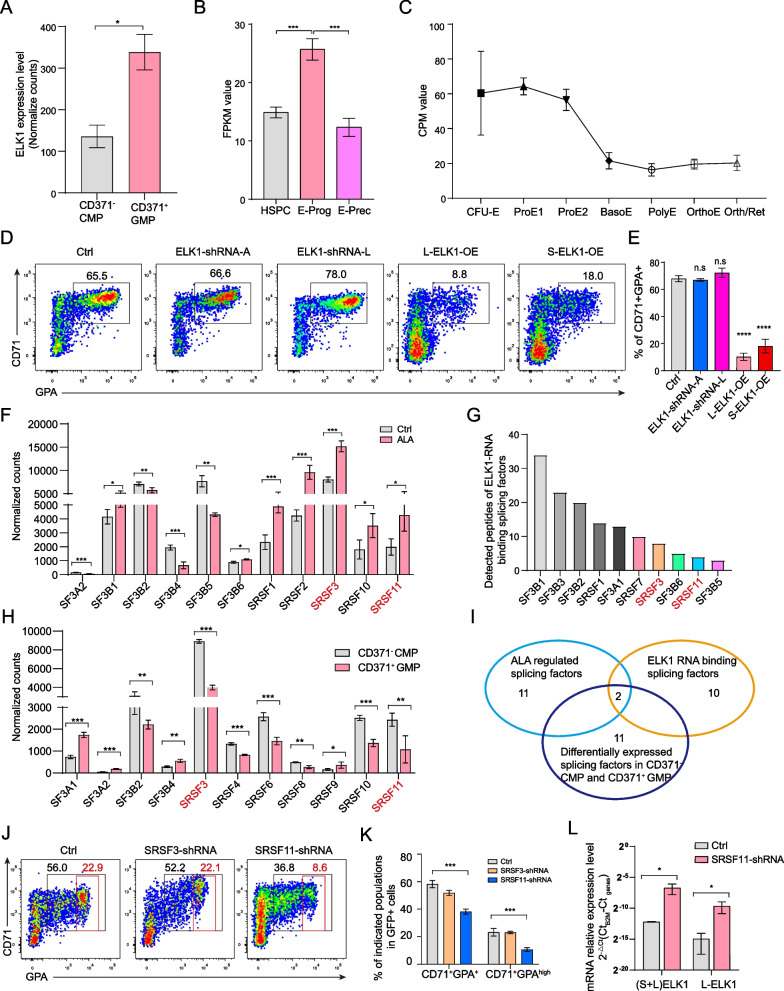
ELK inhibits the erythroid differentiation of CD371^−^ HSPCs in vitro. **A** A bar plot showing the expression level of ELK1 in CD371^–^ CMPs and CD371^+^ GMP. The expression value of ELK1 is shown in normalized counts (normalized by DESeq2 R package) derived from RNA-Seq data. **B** A bar plot showing the expression level of ELK1 in HSPCs, early erythrocyte-committed progenitors (E-Prog), and late erythrocyte-committed progenitors (E-Prec). The expression value of ELK1 is shown in normalized FPKM (Fragments Per Kilobase of exon model per Million mapped fragments) value derived from RNA-Seq data. Data depicted in panels were derived from GSE124164 [35]. **C** A line plot showing the change in ELK1 expression during erythrocyte differentiation. The expression value of ELK1 is shown in normalized CPM (counts per million) value derived from RNA-Seq data. Data depicted in panels were derived from GSE115678 [35, 37]. **D** Cytometry flow assays of CD71^+^GPA^+^ erythrocytes derived from ELK1-shRNA-A, ELK1-shRNA-L, L-ELK1-OE, S-ELK1-OE and Ctrl lentiviral-transduced GFP^+^ CD123^mid^ CD38^mid^ CD371^–^ CD34^+^ CMP-like progenitor cells. The flow cytometry assays were performed on day 12. **E** Statistical analysis of the percentage of CD71^+^ GPA^+^ erythrocytes derived from ELK1-shRNA-A, ELK1-shRNA-L, L-ELK1-OE, S-ELK1-OE, and Ctrl lentiviral-transduced GFP^+^ CD123^mid^ CD38^mid^ CD371^–^ CD34^+^ CMP-like progenitor cells. The percentages were derived from flow cytometry assay on day 12. **F** Bar plots showing the expression level of differentially expressed splicing factors under the treatment of ALA in CD371^+^ GMP. **G** A bar plot showing the ELK1 RNA binding splicing factors. **H** Bar plots showing the expression level of differentially expressed splicing factors in CD371^–^ CMPs and CD371^+^ GMPs. **I** Venn plot showing the splicing factors that upregulated by ALA, bond to ELK1-RNA, and differentially expressed in CD371^–^ CMP and CD371^+^ GMP. **J**–**K** Cytometry flow assays of CD71^+^GPA^+^ erythrocytes derived from SRSF3-shRNA, SRSF11-shRNA lentiviral-transduced GFP^+^ CD123^mid^ CD38^mid^ CD371^–^ CD34^+^ CMP-like progenitor cells. Presentative cytometry plots (J) and statistical analysis results (K) are shown. The flow cytometry assays were performed on day7. **L** A bar plot showing the expression level of (S + L)-ELK1 and L-ELK1 in SRSF11-shRNA and Ctrl-lentiviral transduced CD34^+^ HSPCs detected by RT-PCR. The data in panels (**A**, **B**, **E**, **F**, **H**, **L**) are presented as the mean ± SD. An unpaired Student’s t-test (two-tailed) was performed. N = 3–7 replicates; n.s: no significance, *P < 0.05, ***P < 0.001, ****P < 0.0001

Our previous report shows that ALA targets ELK1 to block the lineage determination of neutrophil [20]. Thus, we hypothesized that ALA also targets ELK1 to regulate erythrocyte differentiation. As previous study shows that CD371^–^ CMP give rise to significantly higher proportion of erythroid colonies than CD371^+^CMPs [36]. To reveal the function of ELK1 in erythrocyte differentiation, we first analyzed the expression level of ELK1 in CD371^–^CMPs and CD371^+^GMPs (Fig. 3A), erythroid progenitors and erythroid precursors (Fig. 3B), and CFU-E to reticulocytes (Fig. 3C) (data was derived from GSE124164 and GSE115678 [35, 37]). We found that expression of ELK1 is lower expression in CD371^–^ CMPs and then down regulated gradually during erythroid differentiation (Fig. 3A–C). The long isoform of ELK1 (L-ELK1) and the short isoform ELK1 (S-ELK1) exert different roles in neutrophil differentiation [20]. We sorted the L-ELK1- and S-ELK1-transduced GFP^+^ CD38^+^ CD123^mid^ CD371^–^ CD34^+^ HSPCs at day 4 from ex vivo cultures for further analysis of differentiation (Additional file 1: Fig. S3A). The result showed that both L-ELK1 and S-ELK1 inhibited erythrocyte differentiation (Additional file 1: Fig. S3B). Further, experiments in which all isoforms of ELK1 were knocked-down (shRNA-A), or only the long isoform was knocked-down (shRNA-L), showed that knocking down the long isoform modestly promoted erythrocyte differentiation (Fig. 3D, E). To further reveal how ALA affect ELK1 to regulate erythrocyte differentiation, we analyzed the ALA regulated splicing factors and the ELK1 RNA binding splicing factors. SRSF3 and SRSF11 were screened out for function assay in erythroid differentiation, which were differentially expressed splicing factors (foldchange > 1.5, p.adj < 0.05) regulated by ALA (Fig. 3F) in CD371^+^GMPs, binding to ELK1 RNA splicing sites (Fig. 3G) and higher expressed in CD371^–^ CMPs compared to CD371^+^ GMPs (Fig. 3H) (Fig. 3I). Knocking down of splicing factors SRSF3 and SRSF11 in CD34^+^CD371^–^ HSPCs showed that knocking down of SRSF11 negatively regulated erythrocyte differentiation (Fig. 3J, K). Furthermore, knocking down of SRSF11 also promoted the differentiation of CD66b^+^ CD15^+^ neutrophils differentiation derived from CD371^+^ GMPs (Additional file 1: Fig. S3C) and upregulated L-ELK1 both at RNA (Fig. 3L) and protein level (Additional file 1: Fig. S3D). In summary, ALA exerts positive regulation on the expression of SRSF11, while SRSF11 exerts negative expression on L-ELK1. Although both L-ELK1 and S-ELK1 regulate neutrophil and erythrocyte differentiation, S-ELK1 has a significantly weaker ability to promote neutrophil differentiation and inhibit erythrocyte differentiation compared to L-ELK1.

### ELK1 promotes myeloid differentiation and inhibits erythrocyte differentiation at early stage

**Fig. 4:**
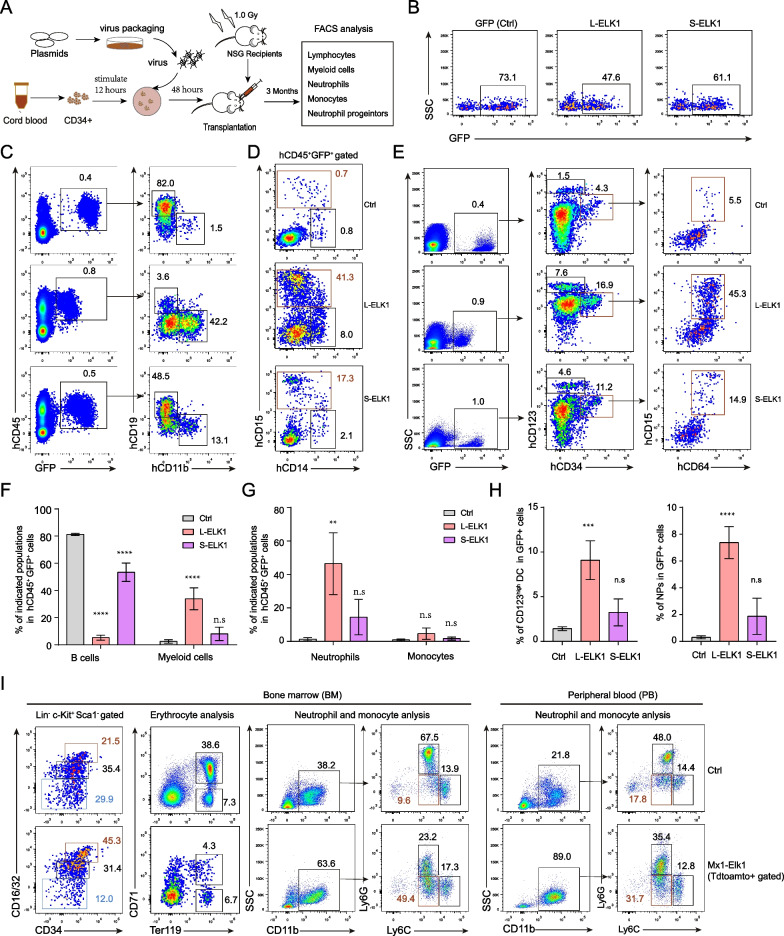
ELK inhibits lymphocyte differentiation and promotes myeloid differentiation. **A** A schematic showing the experimental design used for the analysis of ALA on HSPC lineage differentiation in vivo. **B** Representative flow cytometry plots of pCCL-L-ELK1 (L-ELK1), pCCL-S-ELK1 (S-ELK1), and pCCL-GFP (Ctrl) lentiviral-transduced CD34^+^ HSPCs 48 h after transduction. **C–D** Representative flow cytometry plots of hCD19^+^ B cells, hCD11b^+^ myeloid cells, hCD15^+^ neutrophils, and hCD14^+^ monocytes gated from single hCD45^+^GFP^+^ live cells derived from the bone marrow of M-NSG mice. M-NSG mice were sacrificed 3 months after transplantation. **E** Representative flow cytometry plots of hCD123^high^ dendritic cells (DC) and CD123^mid^ CD34^low/+^ CD15^+^ CD64^mid^ neutrophil progenitors gated from single GFP^+^ live cells derived from the bone marrow of M-NSG mice. M-NSG mice were sacrificed 3 months after transplantation. **F–G** Statistical analysis of hCD19^+^ B cells, hCD11b^+^ myeloid cells, hCD15^+^ neutrophils, and hCD14^+^ monocytes gated from hCD45^+^GFP^+^ single live cells derived from the bone marrow of M-NSG mice. M-NSG mice were sacrificed 3 months after transplantation. **H** Statistical analysis of hCD123^high^ DC and CD123^mid^ CD34^low/+^ CD15^+^ CD64^mid^ neutrophil progenitors gated from single hCD45^+^ GFP^+^ live cells derived from the bone marrow of M-NSG mice. M-NSG mice were sacrificed 3 months after transplantation. **I** Flow cytometry analysis of Lin^–^c-Kit^+^Sca1^–^CD16/32^+^CD34^+^ GMP, Lin^–^c-Kit^+^ Sca1^–^ CD16/32^mid^ CD34^low^ CMP, Lin^–^c-Kit^+^Sca1^–^CD16/32^–^ CD34^–^ MEP, CD71^+^Ter119^+^ erythrocyte progenitors, CD71^–^Ter119^+^ erythrocytes, CD11b^+^ myeloid cells, CD11b^+^ Ly6G^+^ Ly6C^mid^ mature neutrophils, CD11b^+^ Ly6G^–/low^ Ly6C^mid^ immature neutrophils and CD11b^+^ Ly6G^–^ Ly6C^high^ monocytes in bone marrow of Mx1-Elk1and littermate Ctrl mice. Presentative cytometry plots and statical plots are shown. The flow cytometry assays were performed on day7 post the treatment of PolyIC. The data in panels (**F**–**H**) are presented as the mean ± SD. An unpaired Student’s t-test (two-tailed) was performed. N = 3–4 individuals; n.s, no significance; **P < 0.01, ***P < 0.001, ****P < 0.0001

To further validate the impact of overexpression L-ELK1 and S-ELK1 in CD34^+^ HSPCs, we transplanted the L-ELK1 and S-ELK1 overexpressed CD34 + HSPCs into immunodeficiency NSG mice to analyze the changing of hematopoietic lineages and progenitors. (Fig. 4A). We transplanted 20,000 GFP-expressing cells, namely, pCCL-GFP, pCCL-L-ELK1-GFP, and pCCL-S-ELK1-GFP lentiviral-transduced CD34^+^ HSPCs (derived from cord blood), into irradiation-treated NSG mice 48 h after lentivirus transduction (Fig. 4B). CD3^+^ T cells, CD19^+^ B cells, and CD11b^+^ myeloid cells were detected 3 months after transplantation. Significantly lower levels of B cells derived from both L-ELK1- and S-ELK1-transduced CD34^+^ HSPCs, and higher levels of myeloid cells derived from L-ELK1-transduced CD34^+^ HSPCs, were detected in bone marrow (Fig. 4C, F). Further analysis of myeloid cells showed that L-ELK1 significantly increased the level of CD15^+^ neutrophils but did not increase the level of monocytes in bone marrow (Fig. 4D, G). Additionally, we analyzed CD123^high^ dendritic cells (DC) and CD123^mid^ CD34^low/+^ CD15^+^ CD64^+^ neutrophil progenitors [20, 38]. We found that the percentages of CD123^high^ DC cells and CD123^mid^ CD34^low/+^ CD15^+^ CD64^+^ neutrophil progenitors were significantly increased in bone marrow derived from L-ELK1-transduced CD34^+^ HSPCs (Fig. 4E, H). We did not detect CD71^+^ GPA^+^ erythrocytes from the bone marrow of M-NSG mice that only express GM-CSF, because human erythropoiesis in immunodeficient mice is impaired even though the mice express GM-CSF, IL-3, TPO and signal regulatory protein alpha [39], and macrophages prevent human red blood cell reconstitution in immunodeficient mice [40]. Thus, to further validate the impact of Elk1 overexpression in erythropoiesis and granulopoiesis, we constructed a Cre induced Elk1 conditional overexpression mice model (Elk1^LSL/+^) (Additional file 1: Fig. S4A). Mx1-Cre mice was bred with Elk1^LSL/+^ to generate Mx1-Elk1 mice. Flow cytometry assay was performed 7 days post last treatment of PolyIC (Additional file 1: Fig. S4B). The results showed that overexpression Elk1 only modest changed CD3^+^ T cells and CD19^+^ B cells in bone marrow (Additional file 1: Fig. S4C), but greatly increased the GMPs and subsequently increased CD11b^+^ myeloid cells in bone marrow and peripheral blood. At the same time overexpression of Elk1 decreased MEPs and subsequently decreased CD71^+^Ter119^+^, CD71^–^Ter119^+^ erythrocytes in bone marrow (Fig. 4I, Additional file 1: Fig. S4D, E). Furthermore, large number of CD11b^+^Ly6G^low^Ly6C^+^ immature neutrophils were also observed in bone marrow and peripheral blood (Fig. 4I, Additional file 1: Fig. S4F, G). In summary, overexpression of ELK1 promotes myeloid lineages differentiation and inhibits erythroid lineage differentiation at early progenitor stage.

### ELK1 regulates oxygen-related signaling to block erythrocyte differentiation

**Fig. 5:**
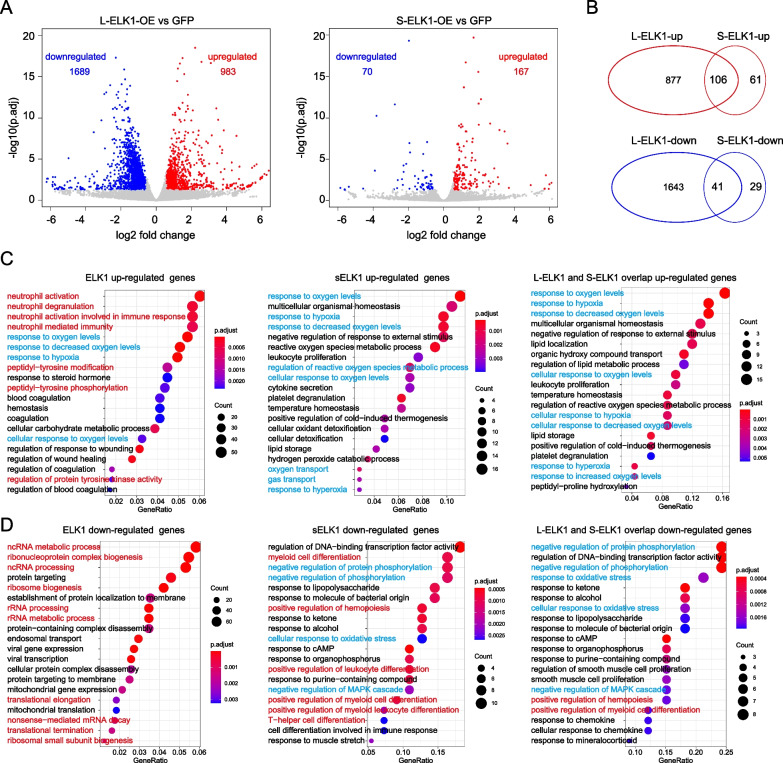
Signals regulated by L-ELK1 and S-ELK1. **A** Volcano plots showing differentially expression genes (fold change > 1.5, p.adj < 0.5) that were upregulated (marked in red) or downregulated (marked in blue) by L-ELK1 and S-ELK1 in GFP^+^ CD123^+^ CD38^+^ CD34^+^ CD371^–^ HPCs. **B** A Venn diagram showing overlapping genes that were upregulated or downregulated by L-ELK1 and S-ELK1 in GFP^+^ CD123^+^ CD38^+^ CD34^+^ CD371^–^ HPCs. **C** GO enrichment analysis of genes that are upregulated by L-ELK1 and S-ELK1 in GFP^+^ CD123^+^ CD38^+^ CD34^+^ CD371^–^ HPCs. **D** GO enrichment analysis of genes that are downregulated by L-ELK1 and S-ELK1 in GFP^+^ CD123^+^ CD38^+^ CD34^+^ CD371^–^ HPCs

L-ELK1 and S-ELK1 are reported to play distinct roles in regulating human neutrophil differentiation [20], while our results showed that overexpression of either L-ELK1 or S-ELK1 inhibited erythrocyte differentiation in vitro (Fig. 3), and B-cell differentiation in vivo (Fig. 4). To further explore the molecular mechanism by which ELK1 regulates erythrocyte differentiation, we performed bulk cell RNAseq of L-ELK1- and S-ELK1-overexpressing CD34^+^ CD123^mid^ CD38^+^ CD371^–^ HSPCs. Differentially expressed genes with a 1.5-fold change and p.adj < 0.05 were filtered (Fig. 5A). To our surprise, we found that only 167 genes were upregulated, and 70 genes were downregulated in S-ELK1-overexpressing HSPCs, which were significantly fewer than the gene expression changes in L-ELK1-overexpressing HSPCs (983 upregulated genes and 1689 downregulated genes) (Fig. 5B). Given that our results showed that both L-ELK1 and S-ELK1 negatively regulated erythrocyte differentiation, we further performed GO enrichment analysis of the genes uniquely upregulated by L-ELK1 or S-ELK1 genes, and the genes upregulated by both L-ELK1 and S-ELK1 (that is, overlapping genes). The results showed that both L-ELK1 and S-ELK1 upregulated oxygen level-related or hypoxia-related signals (Fig. 5C; marked in blue), while L-ELK1 also upregulated both neutrophil activity and tyrosine phosphorylation-related signals (Fig. 5C; marked in red). L-ELK1-downregulated genes were mainly related to noncoding RNA or mRNA processing (Fig. 5D; marked in red), while S-ELK1 downregulated genes were mainly related to protein phosphorylation and oxidative stress-related signals (Fig. 5D; marked in blue) and myeloid differentiation-related signals (Fig. 5D; marked in red).

**Fig. 6:**
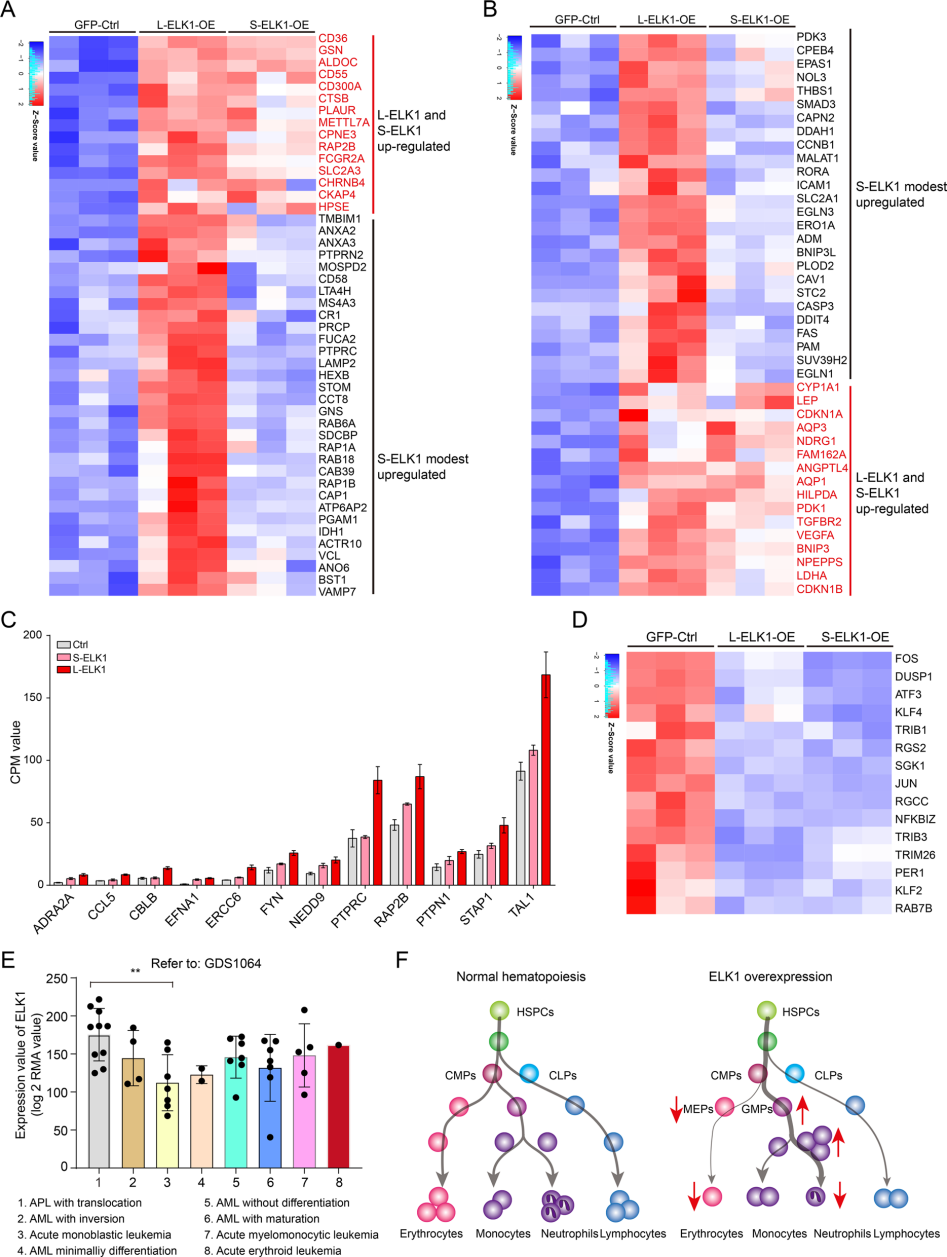
Characteristics of gene expression regulated by L-ELK1 and S-ELK1. **A** A heatmap showing the expression level of neutrophil activity-related genes regulated by L-ELK1 and S-ELK1. **B** A heatmap showing the expression level of hypoxia- and oxidative stress-related genes regulated by L-ELK1 and S-ELK1. **C** Bar plots showing the effect of L-ELK1 and S-ELK1 on the expression of peptidyl-tyrosine phosphorylation (GO:0050730)-related genes. All the expression levels of genes are shown in CPM (counts per million) value derived from RNA-Seq data. **D** A heatmap showing both L-ELK1 and S-ELK1 downregulated genes that negatively regulate phosphorylation (GO:0001933) and positively regulate DNA-binding transcription factor activity (GO:0051090). **E** A bar plot showing the expression value (log2 RMA (Robust Multiarray Average) value detected by Gene Array Scanner (Affymetrix)) of ELK1 in different AML subclasses from the public dataset (GDS1064). **F** A schematic plot showing the global impact of overexpression ELK1 on hematopoiesis

Although L-ELK1, but not S-ELK1, significantly upregulated neutrophil activity-related signals (Fig. 5C), we also found that S-ELK1 significantly upregulated the expression of several neutrophil activity-related genes, including GSN, CTSB, CPNE3 [41], RAP2B [42], FCGR2A [43], HPSE [44], and modestly upregulated ANXA2, ANXA3 [45], MOSPD2 [46], GNS [47], SDCBP [48], PAGM1 [49], HEXB [50], LAPM2 [51], STOM [52], and CR1 [53] (Fig. 6A). Given that both L-ELK1 and S-ELK1 regulated hypoxia- and oxygen-related signals (Fig. 5C), we further analyzed the expression of genes related to hypoxia or oxidative stress signaling. We found that S-ELK1 upregulated several, but not all, of the genes upregulated by L-ELK1 such as CYP1A1 [54], NDRG1 [55], AQP3 [56], ANGPTL4 [57], PDK1 [58], TGFBR2 [59], LDHA [60], BNIP3 [61], and VEGFA [62]. Moreover, the majority of L-ELK1-upregulated genes were moderately upregulated by S-ELK1, including EPAS1 [63], TSP1 [64], SMAD3 [65], DDAH1 [66], RORA [67], CCNB1 [68], ICAM1 [69], EGLN1, EGLN3 [70], ADM [71], BNIP3 [72], PLOD2 [73], CAV1 [74], STC2 [75], and PAM [76] (Fig. 6B). Furthermore, S-ELK1 also moderately upregulated the peptidyl-tyrosine phosphorylation (GO:0050730)-related genes ADRA2A, CCL5, CBLB, EFNA1, ERCC6, FYN, NEDD9, PTPRC, RAP2B, PTPN1, STAP1, and TAL1, which were significantly upregulated by L-ELK1 (Fig. 6C). Both L-ELK1 and S-ELK1 downregulated genes that negatively regulate phosphorylation (GO:0001933) including ATF3, DUSP1, JUN, KLF4, PER1, RGS2, TRIB1, and TRIB3, and downregulated genes related to DNA-binding transcription factor activity (GO:0051090) including FOS, JUN, KLF4, RAB7B, RGCC, SGK1, TRIB1, and TRIM26 (Fig. 6D). Notably, most of these genes that negatively regulate phosphorylation and relate to DNA-binding transcription factor activity play an important role in myelopoiesis. Genes such as FOS [77, 78], KLF4 [79], KLF2 [80, 81], JUN, ATF3 [82], and TRIB3 [83] are involved in the differentiation of erythroid progenitors or erythrocyte differentiation, and genes including RGS2 [84], RAB7B [85], and SGK1 [86] play an important role in megakaryopoiesis, a process that is linked to erythrocyte differentiation. Finally, we also found that ELK1 was high expressed in APL (acute promyelocytic leukemia) compared to Acute monoblastic leukemia (Fig. 6E) derived from the public dataset (GDS1064) [87] and our findings demonstrated that overexpression of ELK1 negatively regulated erythrocyte differentiation while promoted neutrophil lineage differentiation at early stage (Fig. 6F). This suggests that ELK1 might play a significant role as an enhancer in leukemia progression.

## Discussion

Different cell lineages might be regulated by the same molecules but through different signaling pathways. Anemia is usually observed in myeloid leukemias and during the treatment of myeloid leukemias [1, 7, 11, 12, 14]; this observation indicates that genes or pathways exist that regulate both erythropoiesis and myelopoiesis. Identifying the genes or pathways that regulate both erythrocyte and myeloid cell differentiation would help us better understand the relationship between myeloid leukemia and anemia. Previous reports show that ELK1 is essential for neutrophil lineage determination [20] and ELK1 directly regulates human EVI1 gene transcription in acute myeloid leukemia [88].

Here, we show that ALA–ELK1 signals also regulate human erythrocyte differentiation; this finding indicates that there is a link between erythropoiesis and granulopoiesis. ALA and its downstream target ELK1 have multiple functions in human hematopoiesis. It is well known that both erythropoiesis [89] and neutrophil differentiation [90] are regulated by RNA splicing and processing of noncoding RNA. A previous report shows that ALA regulates RNA splicing signals and the splicing of different isoforms of ELK1 [20]. L-ELK1 positively regulates the early differentiation of neutrophils and S-ELK1 positively regulates the terminal differentiation of neutrophils [20]. Here, our data show that both L-ELK1 and S-ELK1 negatively regulate erythrocyte differentiation and knocking down ELK1 RNA binding splicing factor SRSF11 also inhibit erythrocyte differentiation. The surprise is that knocking down of ELK1 does not promote erythrocyte differentiation and ELK1 is differentially expressed in different populations of erythrocyte lineage, which reflect that ELK1 is needed for erythrocyte differentiation and ELK1 exerts different function in different stages of erythrocyte differentiation. Due to both L-ELK1 and S-ELK1 exert positive regulation on neutrophil differentiation and negative regulation in erythrocyte differentiation at early stage, the only difference is that L-ELK1 exerts more stronger regulation function. Thus, the differentiation of neutrophils and erythrocytes at early stage might need to rely on the dose of L-ELK1 and S-ELK1 to achieve precise regulation, which might be regulated by splicing factors. Different splicing factors are differentially expressed in hematopoietic progenitors with different lineage potential, for example SF3A1, SF3B2, SRSF10, SRSF11, SRSF3, SRSF6, SRSF9 are differentially expressed in CD371^+^GMPs neutrophil bias progenitors and CD371^−^ CMPs erythrocyte bias progenitors (Additional file 1: Fig. S5). Totally, precise regulation of L-ELK1 and S-ELK1 is need for neutrophil and erythrocyte lineages differentiation at early stage by different splicing factors.

Hypoxia, hypoxia-induced factors (HIFs; HIF-1, HIF-2, and HIF-3) [91], and phosphorylation [92] play important roles in tumor progression. L-ELK1 upregulates HIFs-related genes such BNIP3 [61], VEGFA [62], EPAS1 [63], TSP1 [64], EGLN1, EGLN3 [70], ADM [71], BNIP3 [72], PLOD2 [73], STC2 [75], AQP3 [56], and PDK1 [58], and the hypoxia-related genes ANGPTL4 [57], TGFBR2 [59], LDHA [60], EOR1A [93], and CCNB1 [68], which are involved in tumor progression. Most of the L-ELK1 downregulates genes involved in the negative regulation of phosphorylation are related to erythropoiesis and megakaryopoiesis. These genes include FOS [77, 78], KLF4 [79], KLF2 [80, 81], JUN, ATF3 [82], and TRIB3 [83], which play an important role in erythroid progenitor or erythrocyte differentiation, and RGS2 [84], RAB7B [85], and SGK1 [86], which play an important role in megakaryopoiesis. These findings indicate that ELK1 might also regulate megakaryocyte differentiation.

Due to the opposite functions of ALA and ELK1 in erythropoiesis and granulopoiesis, blocking the emergence of immature neutrophils or promoting erythrocyte differentiation in neutrophil-related AML would be beneficial for the therapy of neutrophil related AML patients. Given that upregulation of ELK1 will convert erythroid potential CMP into myeloid potential and knocking down of ELK1 only blocks neutrophil without affect erythroid differentiation, we believe that ELK1 could be an important target for therapies that treat myeloid leukemia. Based on our finding that ALA targeting ELK1 to regulate neutrophil and erythrocyte differentiation depends on RNA splicing. Thus, we might deliver shRNA of ELK1 to down regulate the expression of ELK1 to treat neutrophil related leukemia, which would decrease the birth of neutrophils and not affect erythrocytes differentiation. Given that ALA is a natural cell metabolism component of tricarboxylic acid cycle and the metabolism of ALA in patients might limit the therapy efficiency. Some of the modified molecules basing on ALA might be applied to the therapy of leukemias or other cancers by targeting ELK1.

## Conclusions

In this research, we further dissected the new functions of ALA and ELK1 in human granulopoiesis and erythropoiesis. ALA targets ELK1 to regulate human neutrophil and erythrocyte differentiation, which indicates that ALA plus other small molecules might be applied to the generation of functional hematopoietic cells from HPSCs. Overexpression of ELK1 greatly inhibits human erythrocyte differentiation and promotes early neutrophil progenitors’ commitment, while knocking down of ELK1 does not promote erythrocyte differentiation significantly. Totally, ALA-ELK1 signal might be the target of human leukemia therapy.

### Supplementary Information

**Additional file 1.** Supplementary information.

**Additional file 2.** The ARRIVE guidelines 2.0: author checklist.

## Data Availability

All the RNA-Seq data are deposited in NCBI’s Gene Expression Omnibus (GEO) dataset with the accession number GSE217887 and GSE184864. Other data supporting this study and materials are available from the corresponding author upon request.

## Abbreviations

ALA: Alpha lipoic acid
CML: Chronic myeloid leukemia
AML: Acute myeloid leukemia
MDS: Myelodysplastic syndromes
HSC: Hematopoietic stem cell
HPSC: Hematopoietic stem and progenitor cell
HPC: Hematopoietic progenitor cell
GMP: Granulocyte-monocyte progenitor
CMP: Common myeloid progenitor
MEP: Megakaryocyte-erythrocyte progenitor
E-Prog: Erythroid progenitors
E-Prec: Erythroid precursors
CFU-E: Colony forming unit-erythroid
ProE1: Proerythroblasts
ProE2: Proerythroblasts
BasoE: Basophilic erythroblasts
PolyE: Polychromatic erythroblasts
OrthoE: Orthochromatic erythroblasts
Orth/Ret: OrthoE and reticulocytes
DEG: Differential expression gene
ELK1: ETS domain-containing protein Elk-1
SF3B1: Splicing factor 3b, subunit 1
SCF: Stem cell factor
IL-3: Interleukin-3
IL-6: Interleukin-6
TPO: Thrombopoietin Erythropoietin
EPO: Erythropoietin
FLT3L: FMS-like tyrosine kinase-3 ligand
G-CSF: Granulocyte colony stimulating factor
GM-CSF: Granulocyte–macrophage colony stimulating factor
ROS: Reactive oxygen species
CFU: Colony-forming unit
