# Supplementary material for: Small-molecule α-lipoic acid targets ELK1 to balance human neutrophil and erythrocyte differentiation
Source: Stem Cell Res Ther. 2024 Apr 8;15:100. doi: 10.1186/s13287-024-03711-6 (PMC11003016; doi:10.1186/s13287-024-03711-6)
Supplement: Supplementary file 1 — Additional file 1. Supplementary information. [file 13287_2024_3711_MOESM1_ESM.pdf]

Supplementary information

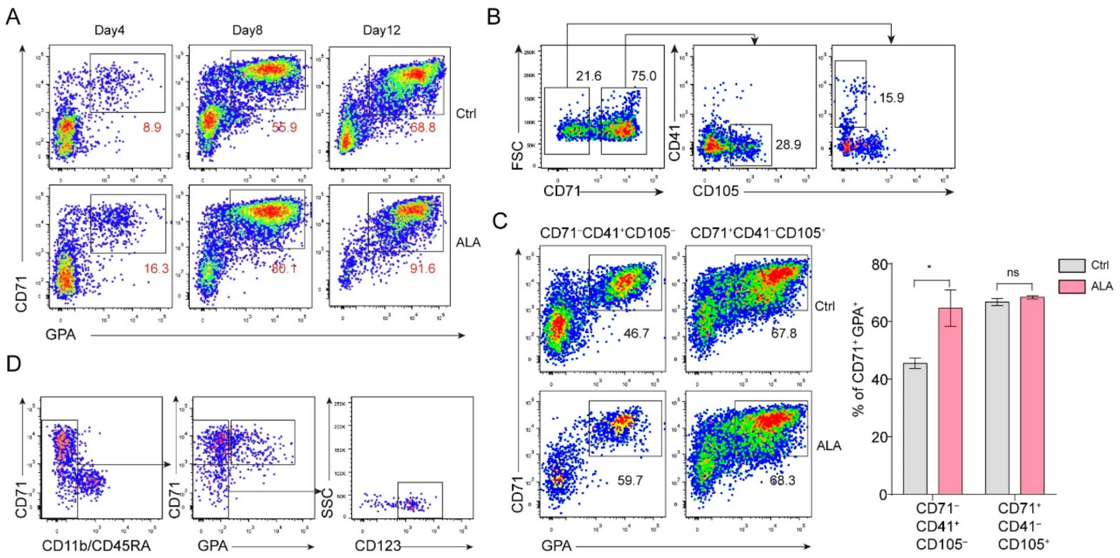

Fig. S1

(A) Representative flow cytometry plots of CD71$^+$ GPA$^+$ erythrocytes derived from CD71$^-$ CD105$^-$GPA$^-$ cells.

(B) Representative sorting gate plots of CD71$^-$ CD41$^+$CD105$^-$ cells and CD71$^+$ CD41$^-$ CD105$^+$ cells derived from the cord blood cultures of CD34$^+$ CD45RA$^-$ HSPCs on day 4.

(C) Flow cytometry analysis of CD71$^+$ GPA$^+$ erythrocytes derived from CD71$^-$ CD41$^+$ CD105$^-$ cells and CD71$^+$ CD41$^-$ CD105$^+$ cells.

(D) Representative sorting gate plots of CD71$^+$GAP$^–$ CFU-E enriched progenitors, CD71$^+$GPA$^{int/low}$ proerythroblast progenitors, and CD11B$^–$ CD45RA$^–$ CD71$^–$ GPA$^–$ CD123^{mid} CMP enriched progenitors derived from CD34$^+$ HSPCs at day 4.

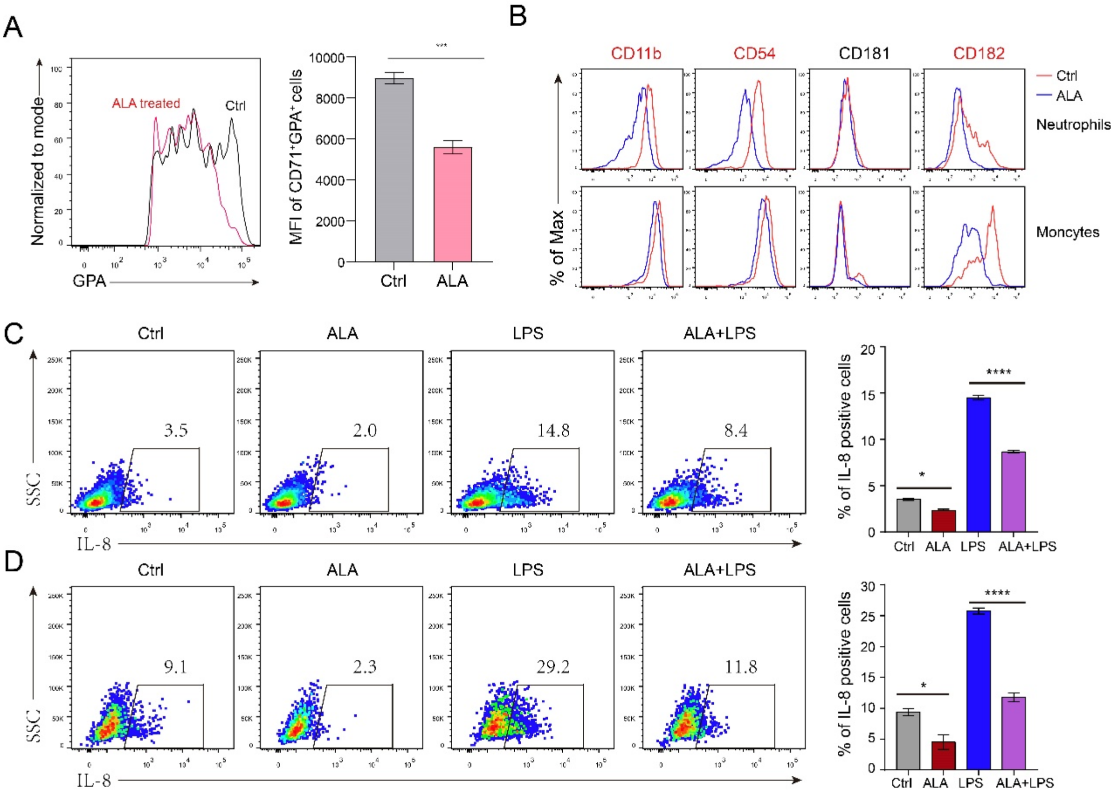

Fig. S2

(A) Mean fluorescence intensity (MFI) analysis of $CD71^+GPA^+$ cells derived from CMPs was performed on day 12 in the presence or absence of ALA.

(B) Flow cytometry analysis showing the expression level of the immune response related surface markers on neutrophils and monocytes derived from $CD34^+$ HSPCs under the treatment of ALA or not for 48 hours.

(C) Flow cytometry analysis showing the expression of IL-8 in neutrophils derived from $CD34^+$ HSPCs under the treatment of ALA or not for 48 hours.

(D) Flow cytometry analysis showing the expression of IL-8 in monocytes derived from $CD34^+$ HSPCs under the treatment of ALA or not for 48 hours. The data in the bar graphs in panel (C, D) are presented as the mean ± SD. An unpaired Student's t-test (2-tailed) was performed. N = 3–5 replicates; $*P < 0.05$, $**P < 0.01$.

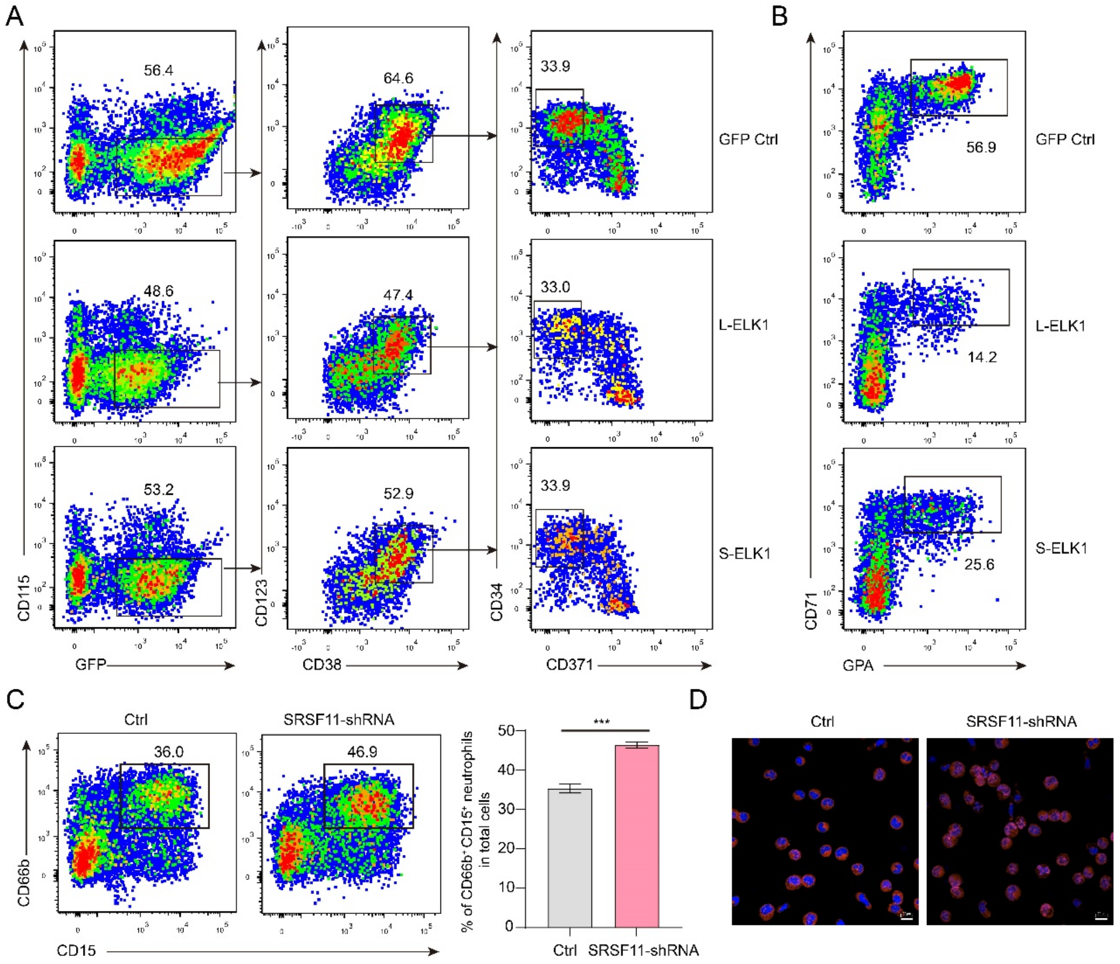

Fig. S3

(A) Sorted gating of L-ELK1, S-ELK1, and Ctrl lentiviral-transduced GFP$^+$ CD123^{mid} CD38^{mid} CD371$^-$ CD34$^+$ CMP-like progenitor cells.

(B) Cytometry flow assay of CD71$^+$GPA$^+$ erythrocytes derived from L-ELK1 S-ELK1 and Ctrl lentiviral-transduced GFP$^+$ CD123^{mid} CD38^{mid} CD371$^-$ CD34$^+$ CMP-like progenitor cells. The flow cytometry assays were performed on day 12.

(C) Cytometry flow assay of CD66b$^+$CD15$^+$ neutrophils derived from SRSF11-shRNA and Ctrl lentiviral-transduced CD371$^+$ GMP cells. The flow cytometry assays were performed on day 8.

(D) Immunofluorescence analysis was performed to measure ELK1 expression level at protein stage in SRSF11-shRNA and Ctrl lentiviral-transduced CD34$^+$ HSPCs at 48 hours. Scale bar = 20 μm.

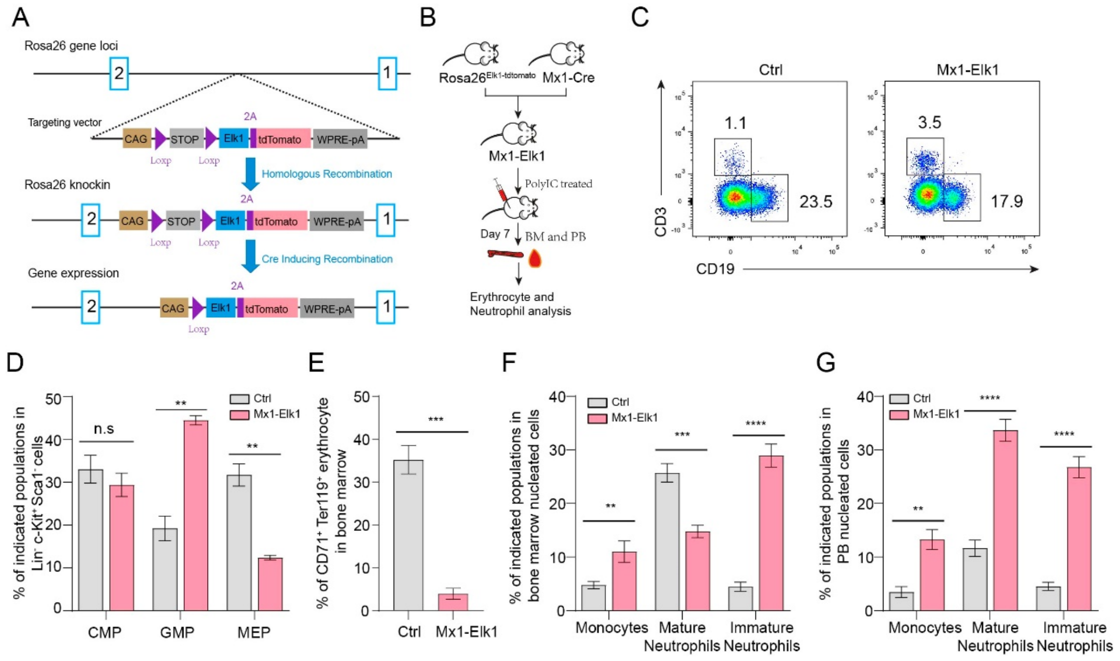

Fig. S4

(A) A schematic plot showing the construction design of Elk1 conditional overexpression mice model.

(B) A schematic plot showing the experiments design to analyze the impact of Elk1 overexpression in mice hematopoiesis.

(C) Presentative flow cytometry plots of $CD3^+$ T cells and $CD19^+$ B cells in the bone marrow of Mx1-Elk1 and littermate Ctrl mice were shown. The flow cytometry assays were performed on day7 post the treatment of PolyIC.

(D) Statical analysis showing the percentages of GMP, CMP and MEP in the bone marrow of Mx1-Elk1 and littermate Ctrl mice. N = 3 individuals; n.s, no significant; $**P < 0.01$, $***P < 0.001$, $****P < 0.0001$.

(E) Statical analysis showing the percentages $CD71^+Ter119^+$ erythrocytes in the bone marrow of Mx1-Elk1 and littermate Ctrl mice. N = 3 individuals; n.s, no significant; $**P < 0.01$, $***P < 0.001$, $****P < 0.0001$.

(F) Statical analysis showing the percentages of $CD11b^+Ly6G^-Ly6C^{high}$ monocytes, $CD11b^+Ly6G^+Ly6C^{mid}$ mature neutrophils and $CD11b^+Ly6G^{low/-}Ly6C^{mid}$ immature neutrophils in the bone marrow of Mx1-Elk1 and littermate Ctrl mice. N = 3 individuals; n.s, no significant; $**P < 0.01$, $***P < 0.001$, $****P < 0.0001$.

(G) Statical analysis showing the percentages of $CD11b^+Ly6G^-Ly6C^{high}$ monocytes,

CD11b^{+}Ly6G^{+}Ly6C^{mid} mature neutrophils and CD11b^{+}Ly6G$^{low/-}$Ly6C^{mid} immature neutrophils in the peripheral blood (PB) of Mx1-Elk1 and littermate Ctrl mice. N = 3 individuals; n.s, no significant; $^{**}P < 0.01$, $^{***}P < 0.001$, $^{****}P < 0.0001$.

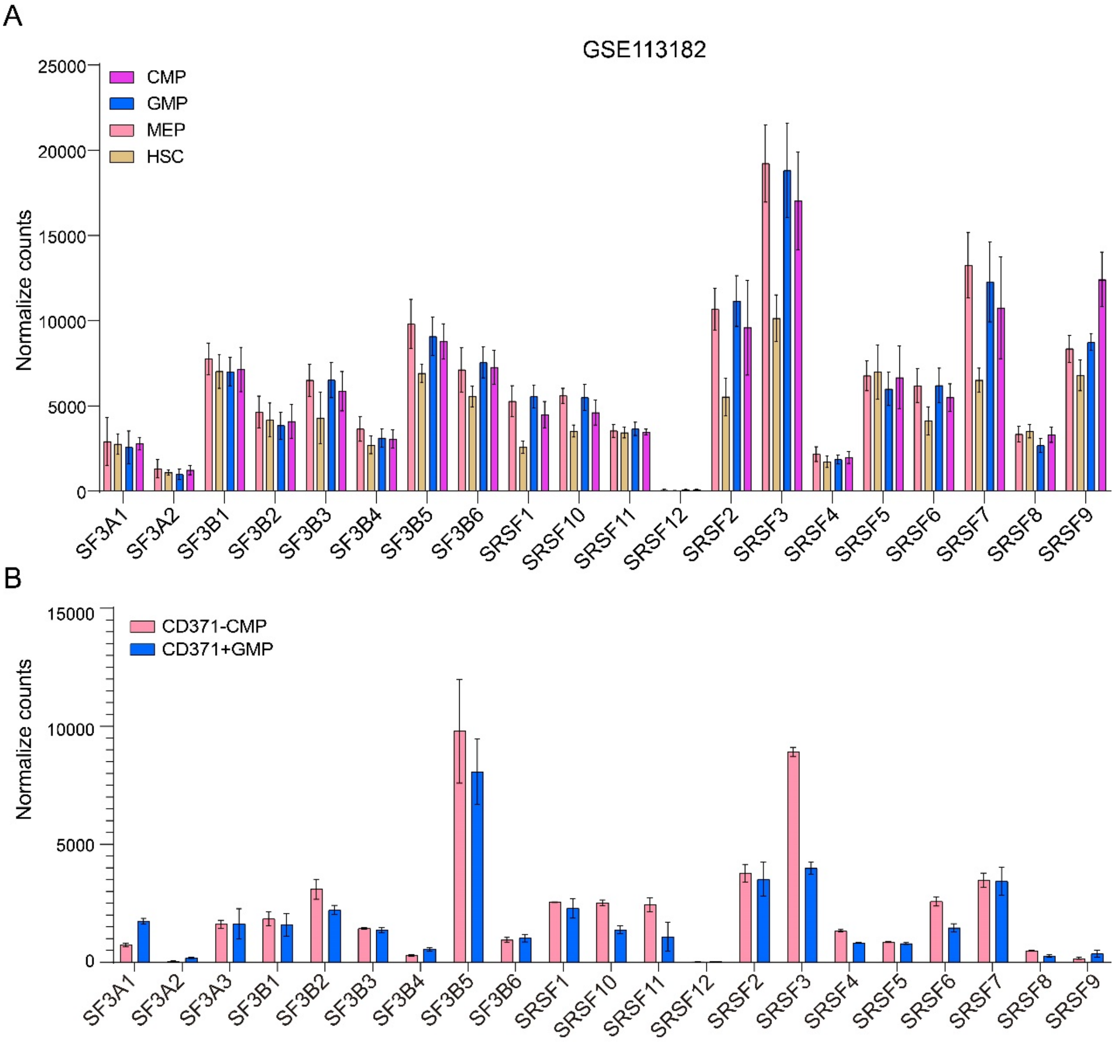

Fig. S5

(A-B) Bar plots showing the expression levels of splicing factors in hematopoietic stem cell (HSC), common myeloid progenitor (CMP), granulocyte-monocyte progenitor (GMP), megakaryocyte-erythrocyte progenitor (MEP) **(A)**, CD371^{-}CMP with and CD371^{+}GMP **(B)**.
